# Ischemic Stroke: New Insights from Risk Factors, Mechanisms and Outcomes

**DOI:** 10.3390/jcdd10120472

**Published:** 2023-11-21

**Authors:** Narayanaswamy Venketasubramanian

**Affiliations:** Raffles Neuroscience Centre, Raffles Hospital, Singapore 188770, Singapore; drnvramani@gmail.com; Tel.: +65-6311-1111

Ischemic stroke (IS) is the most common form of stroke globally [1]. While there are well-known risk factors, there is often a complex interplay of known factors with new ones that places one person at a higher stroke risk compared to another or suggests one mechanistic cause rather than another [2]. Stroke outcomes also vary greatly, from no to severe disability or even death, which may be due to the patient’s pre-morbid status, stroke severity, acute treatment, or environment (e.g., organized stroke care) [3].

Endovascular thrombectomy (EVT) reduces disability if performed early among suitable acute IS patients with symptomatic large vessel occlusion (LVO). A large ischemic core is a contraindication to EVT due to concerns regarding there being little clinical benefit to performing this procedure and the increased risk of harm caused by bleeding. Various commercially available CT-based perfusion (CTP) techniques are able to estimate the ischemic core based on thresholds for cerebral blood volume (CBV) and perfusion by transit times (TTs). Koopman et al. aimed to quantify the volumetric and spatial agreement of the CTP ischemic core estimated with different thresholds and follow-up infarct volume on diffusion-weighted imaging (DWI) at 24 h (contribution 1). In their single-center retrospective study of 55 patients, data were processed with Philips IntelliSpace Portal (Philips Medical Systems, Best, the Netherlands) using four different thresholds resembling what is used in the routine clinical setting among other CTP software packages. The median DWI volume was 10 mL; depending on the threshold used, the median estimated CTP ischemic core volumes ranged from 10–42 mL. In patients with complete reperfusion, the intraclass correlation coefficient (ICC) showed moderate–good volumetric agreement (range 0.55–0.76). However, the correlation was poor if reperfusion was incomplete. Spatial agreement between CTP and DWI was low for all four methods (range 0.17–0.19). Severe core overestimation was most frequently (27%) seen in one of the methods and in patients with terminal carotid occlusion (carotid-T occlusion). CTP may overestimate the final infarct volume shown by DWI at 24 h.

Hyperdense lesions are often seen on brain CT scans after EVT for LVO. These lesions predict subsequent hemorrhage and the final infarct size. They are thought to be due to blood–brain barrier (BBB) disruption, with subsequent leakage and extravasation of contrast administered during the preceding EVT. Whole-brain flat-detector CT (FDCT) is often performed in the angiographic suite immediately after EVT to detect intracerebral hemorrhage. Knott et al. performed a retrospective study on post-EVT FDCT to determine predisposing factors for hyperdense lesions in 474 patients with modified thrombolysis in cerebral infarction score (mTICI) ≥ 2B after EVT indicating successful recanalization (contribution 2). Of the patients, 60.8% had hyperdense lesions. They found that INR (OR 1.21, 95% CI 1.157–1.266, *p* < 0.001), the volume of demarcation (OR 0.997, 95% CI 0.995–0.997, *p* = 0.003), and the FDCT-ASPECTS (OR 0.922, 95% CI 0.883–0.962, *p* < 0.001) were independent factors for the development of a hyperdense lesion.

Among the challenges of EVT is the failure to recanalize and recurrent occlusion. Tirofiban is currently given as a rescue therapy due to its ability to block platelet aggregation and increase the recanalization rate. Due to conflicting efficacy and safety results from trials, Cai et al. investigated the efficacy and safety of tirofiban in a retrospective cohort of 285 patients with LVO with time from onset to puncture (OTP) < 12 h (contribution 3). The decision to administer tirofiban was left with the treating physician. Tirofiban was continuously given at 8 ug/kg/hr after an intravenous bolus of 10 ug/kg if there was no evidence of intracerebral hemorrhage (ICH) on immediate head CT after EVT; 24 h later, dual antiplatelet therapy was given after ICH was ruled out by another head CT. A favorable outcome, defined as a modified Rankin scale (mRS) score of 0–2 at 90 days, was observed significantly more often in patients treated with tirofiban (54.1% vs. 37.9%, *p* = 0.037). There was no significant difference in the safety outcomes of ICH, symptomatic ICH (sICH), or mortality. Subgroup analyses revealed that tirofiban was associated with favorable outcomes in patients with NIHSS > 14 (aOR 2.778, 95% CI 1.056-7.356, *p* = 0.038) but not in patients with NIHSS ≤ 14. No significant heterogeneity was found in the effect of tirofiban across the subgroups of age, sex, ASPECTS, time from onset to puncture, use of t-PA, or stroke etiology.

Randomized controlled trials have shown that intravenous thrombolysis (IVT) reduces disability without increasing mortality among suitable acute IS (AIS) patients treated within 4.5 h of stroke onset. However, patients with end-stage renal disease on hemodialysis (ESRD/HD) are usually excluded from these trials. Egashira et al. performed a narrative review of the benefits and risks of IVT use in AIS patients with ESRD/HD (contribution 4). They searched the electronic database PubMed, reference sections, and additional publications for studies and expert opinions on AIS patients with ESRD/HD on maintenance dialysis that referred to IVT. In total, 10 of 560 studies were included. They found that IVT could improve neurological outcomes and be performed safely despite the increased risk of hemorrhagic complications associated with hypertension. Despite the high complication and mortality rates in ESRD/HD patients with AIS after IVT, the association with IVT was unclear. As for heparin, the study found that experts would administer IVT as long as the aPTT was not more than 1.5 times the baseline level.

A large proportion of patients with LVO remain functionally dependent despite successful reperfusion. Gerbasi et al. built a fully automated pipeline based on a tree ensemble machine learning model to predict poor long-term functional outcomes (mRS 3–6 at 90 days) in 164 AIS patients from the multicenter randomized MR CLEAN-NO IV trial (contribution 5). Data sets containing only imaging (MRI DWI and T2-FLAIR at 24 h), only clinical (including NIH stroke scale score at 24 h), or a combination were analyzed. Including features from both imaging modalities in combination with clinical characteristics led to the best prognostic model (AUC = 0.85, 95% CI 0.81–0.89). The models’ interpretability evaluated using SHapley Additive exPlanations (SHAP) values showed that imaging features from both sequences had a relevant impact on the final classification, with texture heterogeneity of the lesion being the most predictive imaging biomarker.

It is estimated that about 7% of all stroke events occur in hospital, and 1.2% are estimated to occur in the intensive care unit (ICU). Noda et al. provide a narrative review of the risk factors and clinical features of stroke occurring in the ICU (contribution 6). Mechanisms include cardioembolism (e.g., atrial fibrillation (AF), infective endocarditis, manipulations during aortic valve implantation, ascending aortic graft placement, cardiac bypass surgery, percutaneous coronary intervention, implantation or removal of a pacemaker, intra-aortic balloon pumping, extracorporeal membrane oxygenation, and the use of intravascular heart pump devices), after certain non-cardiac surgical procedures (e.g., left upper lobectomy leading to pulmonary vein thrombosis in the long left superior pulmonary vein stump), LVO (e.g., after placement of a stent across the subclavian artery, surgical clipping, and endovascular coiling for intracranial aneurysms), sepsis (leading to AF, systemic inflammation, electrolyte abnormalities, organ dysfunction, and disseminated intravascular coagulation), prothrombotic medications (e.g., erythropoietin- and erythropoiesis-stimulating agents, contraceptives, or hormone replacement therapies), and omitting prescribed anti-thrombotics.

Patients with reduced left ventricular ejection fraction (LVEF) have a higher risk of stroke and mortality as well as poor functional outcomes after stroke. It is unclear if this is also true among those receiving IVT. Chee et al. performed a retrospective study on 937 consecutive AIS patients undergoing thrombolysis in their center (contribution 7). Left ventricular systolic dysfunction (LVSD) was defined as LVEF < 50%, with it affecting 190 patients. They found that LVSD was associated with worse functional mRS outcomes at 3 months (adjusted OR 1.41, 95% CI 1.03–1.92, *p* = 0.030), all-cause mortality (adjusted HR [aHR] 3.38, 95% CI 1.74–6.54, *p* < 0.001), subsequent admission for heart failure (aHR 4.23, 95% CI 2.17–8.26, *p* < 0.001), and myocardial infarction (aHR 2.49, 95% CI 1.44–4.32, *p* = 0.001). LVSD did not predict recurrent stroke/TIA.

Statins (3-hydroxy-3-methylglutaryl-coenzyme A reductase inhibitors) lower low-density lipoprotein cholesterol (LDL-C) levels and the risk of cardio- and cerebrovascular events. They also augment cerebral blood flow, have anti-inflammatory effects, reduce infarct size, and improve neurological function after acute ischemic stroke (AIS). Previous retrospective studies have shown similar or reduced stroke severity with pre-stroke statin therapy; propensity score matching (PSM) studies included serum lipid levels as a confounder. The retrospective PSM study by Mori et al. investigated whether pre-stroke statin use was associated with milder neurological deficits (mND) defined as an NIHSS score ≤3 points at the onset of AIS, without using serum lipid levels at onset (contribution 8). Among the 594 patients with pre-stroke statin use, 308 presented with mND. After PSM, 555 patients received pre-stroke statin treatment, while 286 patients with pre-stroke statin use presented with mND at admission (*p* = 0.0411). The binary-matched pairs contingency table of mND was not symmetrical (*p* = 0.0385). Pre-stroke statin use was associated with mND at the onset of AIS.

Many patients are still disabled after a stroke, despite evidence-based interventions including rehabilitation. This affects their return to their usual pre-stroke level of activity and quality of life. Venketasubramanian et al. used data from a multicenter, randomized, double-blind, placebo-controlled trial of NeuroAiD/MLC601 to analyze how much saving there was in time to functional recovery, defined as an mRS score of 0–1, in patients who received a 3-month oral course of MLC601 (contribution 9). The trial included 548 patients with baseline NIHSS scores of 8–14, an mRS score ≥ 2 at 10 days post-stroke, and at least one mRS assessment at or after month 1. The patients were followed up for 24 months. The authors found that time to functional recovery was significantly shortened for patients receiving MLC601 versus a placebo (log-rank test: *p* = 0.039), with HR 1.30 (0.99–1.70; *p* = 0.059) after adjustment for baseline prognostic factors. On the Kaplan–Meier plot, about 40% achieved functional recovery 18 months earlier in the MLC601 group compared to the placebo (at 6 months versus at 24 months).

Many stroke survivors experience weakness of the upper and lower limbs, which can affect their daily activities of living, mobility, and self-care, especially if the paresis is severe. Bacho et al. studied whether core exercises (trunk exercises) could help stroke survivors to recover motor function if they had very severe motor impairment (contribution 10). They employed a within-subject repeated measures design. Eleven hemiparetic stroke patients aged 24 to 52 years with very severe motor impairment, defined as a Fugl–Meyer assessment (FMA) score < 35, at 3 months post-stroke were enrolled. All participants underwent supervised core exercise training twice weekly for 12 weeks. The main outcome measures were the FMA upper extremity (FMA-UE) and FMA lower extremity (FMA-LE) scores, which were measured before training and at 4-weekly intervals. The authors found significant increases in the mean scores for motor function performance, upper extremity motor function [F(1.416, 12.748) = 28.282, *p* < 0.01, *h*p2 = 0.76], and lower extremity motor function {F(1.676, 34.139) = 32.509, *p* < 0.01, *h*p2 = 0.78) across the 24 weeks.

Among the complications after IS is stroke recurrence. Of concern is the risk of recurrence among drivers, as such an event may incapacitate the driver and lead to accidental injury to said driver, passengers, and others and damage to property. Venketasubramanian et al. retrospectively investigated the frequency and impact of various vascular risk factors on stroke recurrence among 133 drivers referred to their national referral center for the Driving Assessment and Rehabilitation Program (DARP) (contribution 11). Over a median follow-up of 30 months (range 1–78 months), the recurrence rate of stroke was 11.3%, equating to 3.69/100 patient–years. During the completion of multivariable analysis, only age remained as a significant risk factor for recurrence (HR 1.07, 95% CI 1.00–1.13, *p* = 0.045). Among those aged > 60 years, the risk was higher (HR 3.88, 95% CI 1.35–11.20, *p* = 0.012).

Excessive supraventricular ectopic activity (ESVEA), defined in studies as >30 premature atrial contractions (PACs) per hour and/or runs of ≥20 PACs, PAC/hour > 4 and/or supraventricular runs of >5 beats, or >100 PACs per 24 h, has been correlated with the development of AF and is frequently observed in IS patients. Yang et al. searched the PubMed and Embase databases for their meta-analysis of 20 studies that included 23,272 participants (contribution 12). The pooled results showed that ESVEA was associated with an increased risk of AF in the general population (HR 2.57; 95% CI 2.16–3.05), increased risk of AF in ischemic stroke patients (HR 2.91; 95% CI 1.80–4.69), new-onset ischemic stroke (HR 1.91; 95% CI 1.30–2.79), and all-cause mortality (HR 1.41; 95% CI 1.24–1.59). The pooled analysis indicated that ESVEA was not associated with transient ischemic attack (TIA)/recurrent ischemic stroke.

Insertable cardiac monitors (ICMs) allow for continuous long-term electrocardiogram monitoring and the detection of paroxysmal AF (PAF). The standard protocol for removal is via the incision made at the time of implantation, but this may be difficult due to subcutaneous tissue adhesion, device migration from its original location, and capsule formation around the device. Egashira et al. describe an alternative technique for successful ICM removal in 37 cryptogenic stroke patients through an incision directly above the proximal end of the device, perpendicular to the incision made at the time of ICM implantation (contribution 13). The subcutaneous tissue was removed by using forceps along the edge of the proximal end of the ICM. If a capsule was attached to the device, a blade was used to cut the capsule to release the ICM. Once visible, forceps were used to grasp the proximal end of the ICM and the ICM was gently pulled from the pocket. The procedure was a success in all patients. At one week post-operation, there were no intraoperative complications, bleeding, skin ischemia, or wound dehiscence.

Increasing age is the greatest risk factor for stroke. But there are some disadvantages to including it in stroke risk models as age can dominate the risk score and lead to under- or over-predictions in some age groups. Risk factors may contribute differently to the risk of stroke depending on a person’s age. Hunter et al. presented a framework to test if risk factors were affected by age (contribution 14). They then applied the framework to a set of risk factors using Framingham heart study data from the NHLBI Biologic Specimen and Data Repository Information Coordinating Center to determine if there was non-proportionality. Using their framework, they found that diastolic blood pressure, total cholesterol, body mass index (BMI), sex, and high blood pressure treatment may be non-proportional to age. The authors recommend considering testing for the proportionality of risk factors with age in stroke risk prediction modeling.

Efremova et al. described the spectrum of stroke risk factors in a population aged ≥18 years living in the central region of the Republic of Moldova (contribution 15). The study involved 300 subjects, 60% women, with the subjects’ mean age being 49.9 ± 14.5 years. The most common risk factor was abdominal obesity (75% of subjects), general obesity (48%), hypertension (44%), diabetes mellitus (9%), and AF (8%). Left myocardial hypertrophy on ECG was present in 53% of subjects, and acute ischemic changes were present in 2%. Glycosylated hemoglobin was increased by 7%; >50% had dyslipidemia. Homocysteine was increased in 55% of subjects, while high-sensitivity C-reactive protein was seen in 28%.

In summary, recent new knowledge has contributed to a better understanding of ischemic stroke imaging, management, mechanisms, rehabilitation, and risk factors. Various types of perfusion software show moderate–good agreement regarding the estimation of the ischemic core if there is complete reperfusion after endovascular thrombectomy (EVT). Hyperdense lesions on CT after EVT for large vessel occlusion (LVO) are associated with the international normalized ratio (INR), the volume of demarcation, and the flat detector computed tomography Alberta stroke programme early CT score (FDCT-ASPECTS). Intravenous tirofiban after EVT is associated with improved functional outcomes. Long-term poor functional outcomes among LVO patients can be predicted by combining baseline clinical and 24 h MRI data. Intravenous thrombolysis (IVT) improves neurological outcomes among patients with end-stage renal disease on hemodialysis. There are many mechanisms of stroke that occur in the intensive care unit. Left ventricular dysfunction is associated with worse functional outcomes and all-cause mortality after IVT. Pre-stroke statin use is associated with reduced neurological deficit at stroke onset. Approximately 40% of moderately severe patients on MLC601 achieve functional recovery 18 months earlier than those taking a placebo. Core exercises improve motor function among those with severe motor impairment. A history of stroke should not be a contraindication for a return to driving among those otherwise fit to drive. Excessive supraventricular ectopic activity is associated with an increased risk of atrial fibrillation in ischemic stroke patients, new-onset ischemic stroke, and all-cause mortality. Newer and safer techniques are available to remove insertable cardiac monitors from patients suffering from cryptogenic stroke. Testing for the proportionality of risk factors with age is recommended when developing stroke risk prediction models.

The papers highlighted in this Special Issue share new insights into stroke risk factors, mechanisms, and outcomes, offering new knowledge and challenging existing paradigms. Such knowledge will help in improving care and reducing the burden of stroke on the patient, their family, society, and healthcare systems.

## References

[1] GBD 2019 Stroke Collaborators (2021). Global, regional, and national burden of stroke and its risk factors, 1990–2019: A systematic analysis for the Global Burden of Disease Study 2019. Lancet Neurol..

[2] Kuriakose D., Xiao Z. (2020). Pathophysiology and Treatment of Stroke: Present Status and Future Perspectives. Int. J. Mol. Sci..

[3] Hankey G.J. (2017). Stroke. Lancet.

